# Relation of Fibrinogen-to-Albumin Ratio to Severity of Coronary Artery Disease and Long-Term Prognosis in Patients with Non-ST Elevation Acute Coronary Syndrome

**DOI:** 10.1155/2020/1860268

**Published:** 2020-08-17

**Authors:** Mingkang Li, Chengchun Tang, Erfei Luo, Yuhan Qin, Dong Wang, Gaoliang Yan

**Affiliations:** ^1^School of Medicine, Southeast University, Nanjing, Jiangsu, China; ^2^Department of Cardiology, Zhongda Hospital, Southeast University, Nanjing, Jiangsu, China

## Abstract

Previous studies showed that fibrinogen-to-albumin ratio (FAR) regarded as a novel inflammatory and thrombotic biomarker was the risk factor for coronary artery disease (CAD). In this study, we sought to evaluate the relationship between FAR and severity of CAD, long-term prognosis in non-ST elevation acute coronary syndrome (NSTE-ACS) patients firstly implanted with drug-eluting stent (DES). A total of 1138 consecutive NSTE-ACS patients firstly implanted with DES from January 2017 to December 2018 were recruited in this study. Patients were divided into tertiles according to FAR levels (Group 1: ≤8.715%; Group 2: 8.715%~10.481%; and Group 3: >10.481%). The severity of CAD was evaluated using the Gensini Score (GS). The endpoints were major adverse cardiovascular events (MACE), including all-cause mortality, myocardial reinfarction, and target vessel revascularization (TVR). Positive correlation was detected by Spearman's rank correlation coefficient analysis between FAR and GS (*r* = 0.170, *P* < 0.001). On multivariate logistic analysis, FAR was an independent predictor of severe CAD (OR: 1.060; 95% CI: 1.005~1.118; *P* < 0.05). Multivariate Cox regression analysis indicated that FAR was an independent prognostic factor for MACE at 30 days, 6 months, and 1 year after DES implantation (HR: 1.095; 95% CI: 1.011~1.186; *P* = 0.025. HR: 1.076; 95% CI: 1.009~1.147; *P* = 0.026. HR: 1.080; 95% CI: 1.022~1.141; *P* = 0.006). Furthermore, adding FAR to the model of established risk factors, the C-statistic increased from 0.706 to 0.720, 0.650 to 0.668, and 0.611 to 0.632, respectively. And the models had incremental prognostic value for MACE, especially for 1-year MACE (NRI: 13.6% improvement, *P* = 0.044; IDI: 0.6% improvement, *P* = 0.042). In conclusion, FAR was associated independently with the severity of CAD and prognosis, helping to improve risk stratification in NSTE-ACS patients firstly implanted with DES.

## 1. Introduction

Acute coronary syndrome (ACS) is today one of the leading causes of morbidity and mortality in the world. Despite of the use of current guideline-recommended therapies, including prompt coronary revascularization, dual antiplatelet therapy, and intensive lipid-lowering therapy, ACS patients still have a poor prognosis [1–3]. Therefore, early risk stratification is essential for making clinical decision and evaluating prognosis.

ACS is a group of clinical syndromes, including non-ST elevation acute coronary syndrome (NSTE-ACS) and ST elevation acute myocardial infarction (STEMI), with the pathogenesis of vulnerable plaque rupture, subsequent red or white thrombosis, thereby resulting in incomplete or complete vascular occlusion [4]. The typical plaque is characterized by a large central lipid core, an abundance of inflammatory cells, a thin fibrous cap, and a paucity of smooth muscle cells [5]. Previous study noted that inflammatory response was a key regulator in the degradation of extracellular collagen, which in turn led to the thinning of fiber cap [6]. Simultaneously, blood stasis, endothelial damage, and hypercoagulability were also considered risk factors for acute coronary thrombotic events [7].

Recently, numerous studies have shown that the fibrinogen-to-albumin ratio (FAR) does not only have a high prognostic value in certain types of cancer including gastric, colorectal, and gallbladder cancer [8–10] but also have an association with the presence and poor clinical outcome of cardiovascular disease [11–14]. However, none has addressed the association between FAR and severity of coronary artery disease (CAD), clinical outcome in NSTE-ACS patients firstly implanted with drug-eluting stent (DES). Thus, the aim of the present study is to investigate the relationship between FAR and severity of CAD measured by the Gensini Score (GS), and the prognostic value of FAR in predicting long-term prognosis in NSTE-ACS patients firstly implanted with DES.

## 2. Materials and Methods

### 2.1. Study Population

**Figure 1:**
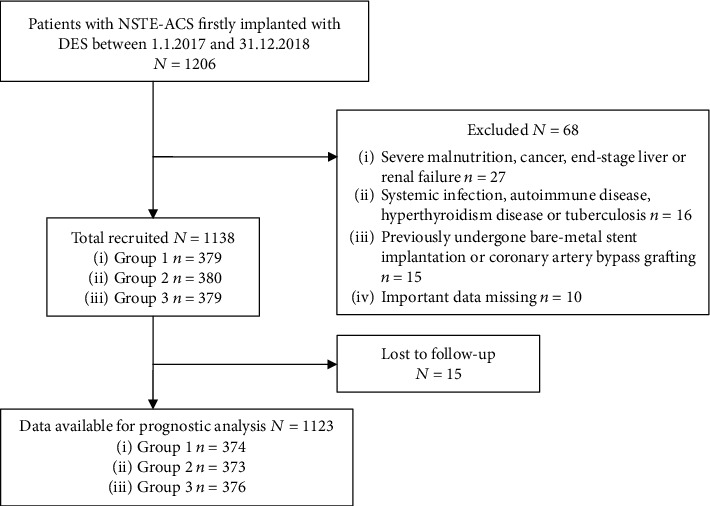
Flow diagram of participant selection.

The present investigation (Figure 1) was a single-center, observational, prospective cohort study among 1206 consecutive NSTE-ACS patients firstly implanted with DES at Department of Cardiology, Zhongda Hospital, Jiangsu, China, from January 2017 to December 2018. The diagnosis of NSTE-ACS was based on the criteria determined by the American College of Cardiology [1]. Patients with severe malnutrition, cancer, end-stage liver or renal failure, systemic infection, autoimmune disease, hyperthyroidism disease, and tuberculosis were excluded from this study. Patients who have previously undergone bare-metal stent implantation or coronary artery bypass grafting were also excluded. Moreover, patients with important data missing were excluded as well from the study. Finally, 1138 patients remained in the study sample. With the 33.33th and 66.67th percentile of FAR being 8.715% and 10.481%, respectively, the patients were accordingly divided into 3 groups as follows: Group 1 (FAR ≤ 8.715%, *n* = 379), Group 2 (8.715% < FAR ≤ 10.481%, *n* = 380), and Group 3 (FAR > 10.481%, *n* = 379). All patients provided written informed consent before enrollment in the study.

### 2.2. Data Collection

The baseline and clinical characteristics (including demographic data, previous medical history, vital signs on admission, laboratory indices, echocardiography results, angiographic variables, and medication use at discharge) were gathered from the medical record systems. Smoker is defined as the person with continuous or cumulative smoking for at least 6 months in a lifetime. Body mass index (BMI) was calculated as the body mass divided by the square of the height and presented in units of kg/m^2^. The venous blood samples were obtained from each participant within 24 hours of admission at room temperature. All laboratory indices were uniformly and preoperatively performed by the Department of Laboratory Medicine, Zhongda Hospital. The FAR was calculated by using the following formula: serum fibrinogen (g/L)/albumin (g/L)∗100%. Echocardiography was performed to record left ventricular ejection fraction (LVEF), which was used to evaluate the left ventricular systolic function.

### 2.3. Coronary Interventions and Medications

All patients were operated by two cardiologists specialized in intervention treatment. The corresponding diseased vessels were implanted with DES according to the specific results of coronary angiography. Coronary lesions leading to diameter stenosis in vessels were scored separately and added together to provide the cumulative GS in accordance with the Gensini Score system [15]. Severe CAD was defined as GS > 60. After operation, the medications such as aspirin, clopidogrel/ticagrelor, beta blockers, statins, angiotensin-converting enzyme inhibitors (ACEI), or angiotensin receptor blocker (ARB) were commonly used according to clinical conditions.

### 2.4. Endpoint and Follow-Up

The endpoints were major adverse cardiovascular events (MACE) including all-cause mortality, myocardial reinfarction, and target vessel revascularization (TVR) during the follow-up period (30 days, 6 months, and 1 year after first DES implantation). All participants were followed up by telephone.

### 2.5. Statistical Analysis

Data analysis was performed using SPSS 22.0 (SPSS Inc. Chicago IL, USA) and R version 4.0.2 (http://www.r-project.org). Continuous variables with normal distribution were expressed as the mean ± SD and compared using variance analysis. Continuous variables without normal distribution were expressed as the median (25th-75th interquartile range) and compared using the Kruskal-Wallis *H* test. Categorical variables were reported in frequencies with percentages and compared using Chi-square test. Spearman's correlation coefficient was used to examine the correlations of FAR with troponin I (TnI), creatine kinase-MB (CK-MB), and GS. Cumulative incidences were graphically represented using the Kaplan-Meier curves, and the differences in cumulative incidences were compared using the log-rank test. Univariate logistic regression analysis was used to evaluate the predictors of severe CAD. Multivariate logistic regression analysis was performed to identify the independent predictors of severe CAD. Univariate and multivariate Cox proportional hazard's models were employed to examine whether FAR was an independent endpoint predictor. Variables with *P* < 0.10 on univariate analysis were selected for the multivariate analysis. The results were expressed as hazard ratio (HR) with the corresponding 95% confidence interval (CI). Multivariate Cox regression analysis was performed using a backward stepwise method. The predictive performances of established risk factors, FAR, and the established risk factors combined with FAR were assessed by C-statistic. Moreover, absolute integrated discrimination improvement (IDI) and net reclassification improvement (NRI) were used to evaluate improvements in risk prediction quantification. All tests were 2-tailed, and *P* < 0.05 was considered statistically significant.

## 3. Results

### 3.1. Baseline and Clinical Characteristics

**Figure 2:**
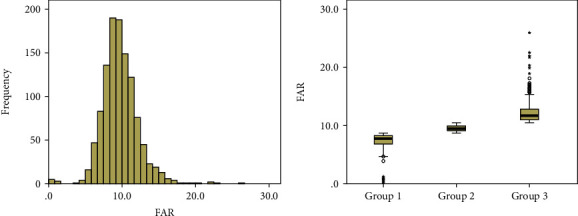
Statistical histogram and boxplot display the distribution of FAR among the study cohort.

A total of 1138 NSTE-ACS patients were included in this study, including 429 females (37.7%). The study population had an average age of 66.5 ± 10.5 years, and a median FAR of 9.7% (8.3%, 11.0%). Statistical histogram and boxplot of FAR are given in Figure 2. Baseline and clinical characteristics are summarized in Table 1. There were statistically significant differences among the three groups in terms of the age, female, diabetes mellitus, leukocyte count, hemoglobin, platelet count, D-dimer, TnI, glucose, creatinine, uric acid, FAR, LVEF, clinical presentation, three-vessel disease, long lesions, calcified lesions, and GS (*P* < 0.05). No statistically significant difference was found in the other indicators. Patients with elevated FAR level tended to be older and have higher prevalence of diabetes mellitus. Leukocyte count, D-dimer, TnI, glucose, and creatinine were also higher in patients with elevated FAR level, while the concentration of hemoglobin showed an opposite trend (*P* < 0.001). Meanwhile, patients with a high FAR had more complicated coronary artery lesion: higher incidences of three-vessel disease, long lesions, and calcified lesions (*P* < 0.05) and higher GS (*P* < 0.001).

### 3.2. Correlations of FAR with TnI and CK-MB

**Figure 3:**
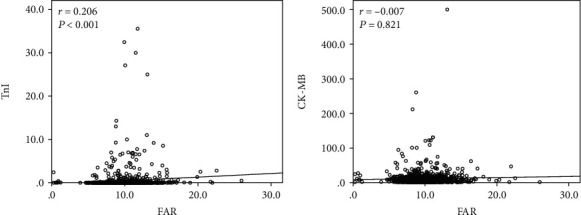
Correlations of FAR with TnI and CK-MB.

The correlations of FAR with TnI and CK-MB are shown in Figure 3. FAR was positively correlated with TnI (*r* = 0.206, *P* < 0.001). However, no statistically significant correlation was observed between FAR and CK-MB (*r* = −0.007, *P* = 0.821).

### 3.3. The Relationship between FAR and Severity of CAD

**Figure 4:**
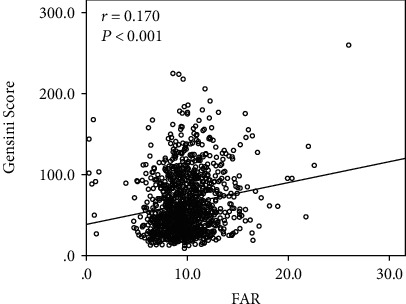
Scatter dot displays the relationship between FAR and GS.

Spearman's correlation analysis revealed that there was significantly positive correlation between FAR and GS (*r* = 0.170, *P* < 0.001, Figure 4). After adjusting for age, female, TnI, BMI, hypertension, diabetes mellitus, leukocyte count, hemoglobin, glucose, creatinine, uric acid, LVEF, and NSTEMI, multivariate logistic regression analysis reported that high FAR was an independent risk factor for patients with severe CAD (OR: 1.060; 95% CI: 1.005~1.118; *P* < 0.05, Table 2).

### 3.4. Risk Factors for MACE

**Figure 5:**
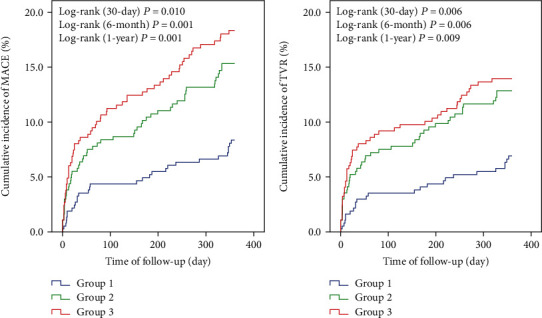
The Kaplan-Meier analysis curve of MACE and TVR among 3 groups.

Eventually, 1123 patients completed the clinical follow-up, and the follow-up rate reached at 98.7%. The postoperative follow-up for each group is shown in Table 3. A total of 55, 98, and 146 cases, respectively, developed MACE during the follow-up period (30 days, 6 months, and 1 year after first DES implantation). The cumulative incidences of MACE and TVR increased significantly with higher tertile of FAR, demonstrating that elevated FAR level was associated with poor cardiovascular outcomes (log-rank *P* < 0.05; Figure 5).

The results of Cox regression analysis are presented in Table 4. Univariate Cox regression analysis showed that FAR was a risk factor for MACE within 30 days, 6 months, and 1 year after DES implantation. After adjusting for potential confounding factors (for 30-day MACE: female, BMI, diabetes mellitus, leukocyte count, CK, CK-MB, creatinine, and NSTEMI; for 6-month MACE: female, BMI, leukocyte count, APTT, TnI, CK, CK-MB, creatinine, NSTEMI, and beta blockers; and for 1-year MACE: female, BMI, hypertension, leukocyte count, TnI, CK, CK-MB, glucose, creatinine, LVEF, NSTEMI, and beta blockers), multivariate Cox regression analysis showed that FAR was an independent predictor for MACE at 30 days, 6 months, and 1 year after DES implantation (HR: 1.095; 95% CI: 1.011~1.186; *P* = 0.025. HR: 1.076; 95% CI: 1.009~1.147; *P* = 0.026. HR: 1.080; 95% CI: 1.022~1.141; *P* = 0.006), while BMI and NSTEMI were likewise independent predictors for MACE in NSTE-ACS patients firstly implanted with DES.

The evaluation of predictive models for MACE is shown in Table 5. The C-statistics of FAR for predicting 30-day, 6-month, and 1-year MACE were 0.628 (95% CI: 0.560~0.696), 0.609 (95% CI: 0.556~0.661), and 0.593 (95% CI: 0.549~0.637), respectively. Adding FAR to the models of established risk factors, the C-statistic increased from 0.706 to 0.720, 0.650 to 0.668, and 0.611 to 0.632, respectively. These results suggested that the prognostic performance of established risk factors + FAR was better than that of FAR or established risk factors. Moreover, with the addition of FAR, the models had incremental prognostic value for MACE, especially for 1-year MACE (NRI: 13.6% improvement, *P* = 0.044; and IDI: 0.6% improvement, *P* = 0.042).

## 4. Discussion

In this study, a prospective analysis of 1138 consecutive NSTE-ACS patients firstly implanted with DES was performed. The results showed that FAR was significantly and positively correlated with the GS, a scoring system used to determine the severity of CAD, and elevated FAR was an independent risk factor for severe CAD. In addition, the Kaplan-Meier curve showed that the accumulative incidences of MACE and TVR in the 3 groups progressively increased with rising levels of FAR. After adjusting for the confounding factors, FAR was an independent predictor for MACE at 30 days, 6 months, and 1 year after DES implantation (HR: 1.095; 95% CI: 1.011~1.186; *P* = 0.025. HR: 1.076; 95% CI: 1.009~1.147; *P* = 0.026. HR: 1.080; 95% CI: 1.022~1.141; *P* = 0.006, respectively). If other factors remained unchanged, for each 1% increase in FAR, the risks of MACE increased by 9.5%, 7.6%, and 8.0%, respectively. Moreover, with the addition of FAR, the prognostic performance of the model was improved. Taken together, these findings suggested that FAR had a great clinical value in NSTE-ACS patients firstly implanted with DES. And it may be worth to monitor FAR, which would identify the NSTE-ACS patients at high risk.

Fibrinogen is a serum glycoprotein with a dimeric molecular structure synthesized by the liver [16], which plays an important role in the inflammatory and coagulation cascade. Previous studies confirmed that by upregulating the expression of proinflammatory cytokines including tumor necrosis factor-*α*, interleukin-1, and interleukin-6 or promoting the activation and adhesion of macrophage, fibrinogen helped to strengthen systemic or local vascular inflammation, secondary vascular endothelial damage, then subendothelial aggregation and oxidation of low-density lipoprotein, and finally the proliferation and migration of vascular smooth muscle cells [17, 18]. All of these reactions eventually led to the formation and vulnerability of atherosclerotic plaque [4, 19]. Furthermore, it was reported that blood viscosity and peripheral resistance increased with plasma fibrinogen levels, resulting in blood oxygen transport disorder, slow blood flow, and red blood cell aggregation, thereby increasing the risk of thrombosis [20, 21]. In addition, fibrinogen might accelerate platelet aggregation by binding to platelet surface GPIIb/IIIa receptors [22]. As coagulation factor I, fibrinogen degrades to cross-linked fibrin polymer (a part of thrombus) with the help of thrombin and factor XIIIa [16]. The study by Peng et al. showed that admission of fibrinogen ≥ 3.17 g/L was an independent predictor of all-cause and cardiac mortality in patients with CAD [23]. Verdoia et al. reported that high fibrinogen level was an independent predictor of the presence and severity of CAD [24].

Serum albumin, the main component of maintaining plasma colloid osmotic pressure, is also involved in acute inflammatory reactions. Physiological level of serum albumin inhibits the expression of vascular cell adhesion molecule-1 and increases the elimination of oxygen-free radicals, thereby reduces the inflammatory response and endothelial cell apoptosis, suggesting albumin to be an anti-inflammatory and antioxidant factor [25–27]. Rezkalla et al. showed that hypoproteinemia might aggravate ischemia and reperfusion injury in patients with coronary no-reflow phenomenon [28]. And albumin may also inhibit platelet activation and aggregation by promoting the expression of prostacyclin D2 and inhibiting thromboxane synthase activity [29]. Additionally, hypoalbuminemia causes high blood viscosity by increasing red cell lysophosphatidylcholine, leading to endothelial dysfunction [30]. More interestingly, serum albumin is inversely correlated with fibrinogen. Hypoalbuminemia stimulates the synthesis of lipoprotein and procoagulant factors such as factor V, factor VIII, and fibrinogen as a compensatory response, which results in hyperlipidemia and hypercoagulability, eventually promoting atherosclerotic plaque and thrombosis [31]. A study by Phillips et al. suggested that serum albumin was associated with mortality from cardiovascular disease [32]. Oduncu et al. showed that serum albumin levels on admission had high prognostic value in patients with STEMI undergoing percutaneous coronary intervention (PCI) [33].

Covering both fibrinogen and albumin, FAR reflected systemic inflammation in patients with rheumatoid arthritis, which was comparable to C-reactive protein [34]. Besides, FAR had higher specificity and sensitivity than fibrinogen in predicting the severity of chronic venous insufficiency, a progressive inflammatory disease [35]. Furthermore, some studies [10, 36] reported that the discriminatory power of FAR for prognosis was obviously superior to that of the albumin and fibrinogen in tumor patients, possibly with the fact that systemic inflammatory response and hypercoagulation state are critically involved in the progression of human malignancies. Hence, FAR was concluded to be a better biomarker of inflammation and coagulation. In fact, previous studies have demonstrated that FAR was associated with the severity of CAD and adverse outcome in STEMI patients. Karahan et al. showed that FAR was significantly related to SYNTAX score in predicting the severity of CAD in patients with STEMI [37]. Zhao et al. showed that admission FAR had a high sensitivity and specificity for identifying angiographic no-reflow and short-term mortality in patients with STEMI undergoing primary PCI [14]. Xiao et al. showed that preoperative FAR was positively correlated with C-reactive protein, GRACE scores, and mortality, suggesting that FAR could serve as a prognostic indicator in STEMI patients undergoing primary PCI [13]. However, few studies have focused on the clinical value of FAR in NSTE-ACS patients. Recently, He et al. showed that a high FAR was an independent predictor of 1-year all-cause mortality in patients with NSTE-ACS undergoing PCI (HR: 2.223; 95% CI: 1.002~4.931; *P* = 0.049), although the *P* value is close to 0.05 [11]. In the present study, there was no significant difference in the incidence of all-cause mortality among 3 groups. This may be linked to the low incidence of all-cause mortality. To supplement, our study indicated that FAR was positively correlated with the severity of CAD and increased risk of MACE, TVR in NSTE-ACS patients at 30 days, 6 months, and 1 year after first DES implantation, suggesting FAR could be used to be a potential prognostic indicator. Moreover, with the addition of FAR, the models had incremental prognostic value for MACE. To the best of our knowledge, this is the first study to report the correlation among FAR, severity of CAD, and clinical outcome in patients with NSTE-ACS who firstly underwent DES implantation.

The following limitations of this study should be addressed. First, this study is a single-center study; therefore, there may be selection bias when patients are admitted to the hospital, and the follow-up time might not be long enough. Second, traditional inflammatory factors such as C-reactive protein are not involved in the present study. Furthermore, since the FAR was only measured on admission, no further analysis of FAR during the follow-up period was performed. In addition, the lack of intravascular ultrasonography in the study subjects may affect the judgment of subclinical atherosclerosis in coronary arteries. Finally, this study is not a randomized controlled study. Large-scale randomized controlled studies are still needed to further evaluate the predictive value of FAR on the severity of CAD and prognosis in NSTE-ACS patients.

## 5. Conclusions

FAR was significantly related to GS in predicting the severity of CAD and proved to be an independent risk factor for severe CAD. Simultaneously, FAR was an independent predictor for MACE in NSTE-ACS patients at 30 days, 6 months, and 1 year after first DES implantation, respectively. And FAR could help to improve the prognostic performance of the established risk factors as well. Thus, the FAR may serve as a convenient, effective, and noninvasive biomarker to indicate the severity of CAD, to predict the prognosis, and to improve risk stratification in NSTE-ACS patients firstly implanted with DES.

## Figures and Tables

**Table 1:** Baseline and clinical characteristics.

Variable	Group 1 (*n* = 379)	Group 2 (*n* = 380)	Group 3 (*n* = 379)	*P* value
FAR, %	7.8 (6.8, 8.2)	9.5 (9.1, 9.9)	11.7 (11.0, 12.8)	<0.001
Age, years	64.2 ± 10.1	66.8 ± 10.2	68.5 ± 10.6	<0.001
Female	117 (30.9%)	161 (42.4%)	151 (39.8%)	0.003
BMI, kg/m^2^	25.0 ± 3.1	24.8 ± 3.3	25.0 ± 3.3	0.606
SBP, mmHg	136.7 ± 19.8	136.8 ± 19.6	136.1 ± 20.7	0.881
DBP, mmHg	77.3 ± 12.4	76.4 ± 12.5	76.1 ± 13.4	0.401
Hypertension	267 (70.4%)	280 (73.7%)	281 (74.1%)	0.461
Diabetes mellitus	102 (26.9%)	127 (33.4%)	136 (35.9%)	0.024
Smoker	106 (28.0%)	105 (27.6%)	94 (24.8%)	0.557
Family history of CAD	11 (2.9%)	7 (1.8%)	5 (1.3%)	0.288
History of cerebrovascular disease	88 (23.2%)	80 (21.1%)	91 (24.0%)	0.603
Atrial fibrillation	28 (7.4%)	27 (7.1%)	33 (8.7%)	0.678
Biochemical indicators				
Leukocyte count, 10^9^/L	6.2 (5.3, 7.4)	6.5 (5.5, 7.8)	7.0 (5.8, 8.6)	<0.001
Hemoglobin, g/L	142 (132, 152)	137 (127, 147)	132 (120, 143)	<0.001
Platelet count, 10^9^/L	196 (161, 233)	195 (163, 235)	208 (170, 256)	0.001
PT, s	11.6 ± 2.9	11.4 ± 1.2	11.7 ± 1.6	0.152
APTT, s	31.0 ± 4.8	30.7 ± 5.7	31.2 ± 5.0	0.446
D-dimer, *μ*g/L	76 (43, 131)	103 (61, 169)	145 (88, 256)	<0.001
TnI, ng/mL	0 (0,0.01)	0 (0,0.02)	0.01 (0,0.10)	<0.001
CK, U/L	87.0 (65.3,128.0)	83.0 (56.3,122.8)	87.0 (56.3,136.0)	0.084
CK-MB, ng/mL	8.8 (3.4,12.9)	8.9 (4.2,13.5)	8.2 (3.2,13.8)	0.597
Glucose, mmol/L	5.8 (5.2, 7.0)	5.9 (5.3, 7.4)	6.0 (5.2, 8.2)	0.034
Creatinine, *μ*mol/L	75 (64, 87)	76 (66, 89)	80 (68, 97)	<0.001
Uric acid, *μ*mol/L	353 (296, 412)	343 (285, 420)	374 (299, 438)	0.003
Triglycerides, mmol/L	1.5 (1.0, 2.2)	1.4 (1.0, 2.0)	1.4 (1.0, 2.0)	0.186
Total cholesterol, mmol/L	4.5 ± 1.2	4.5 ± 1.2	4.5 ± 1.2	0.759
LDL-C, mmol/L	2.7 ± 1.0	2.7 ± 1.0	2.8 ± 1.0	0.516
Echocardiography				
LVEF, %	67 (63, 72)	67.4 (62.0, 72.0)	65.2 (58.0, 72.0)	0.032
Clinical presentation				<0.001
Unstable angina	319 (84.2%)	314 (82.6%)	253 (66.8%)	
NSTEMI	60 (15.8%)	66 (17.4%)	126 (33.2%)	
Coronary angiography				
Number of stents	1.5 ± 0.7	1.5 ± 0.7	1.5 ± 0.7	0.753
Left main disease	23 (6.1%)	29 (7.6%)	36 (9.5%)	0.209
Three-vessel disease	130 (34.3%)	154 (40.5%)	188 (49.6%)	<0.001
Long lesion	181 (47.8%)	198 (52.1%)	215 (56.7%)	0.047
Calcified lesion	55 (14.5%)	80 (21.1%)	92 (24.3%)	0.003
Gensini Score	48 (31, 75)	52 (32, 87)	63 (42, 96)	<0.001
Medication use at discharge				
Aspirin	370 (97.6%)	372 (97.9%)	375 (98.9%)	0.362
Clopidogrel/ticagrelor	378 (99.7%)	380 (100%)	378 (99.7%)	0.555
ACEI/ARB	192 (50.7%)	216 (56.8%)	222 (58.6%)	0.070
Beta blockers	284 (74.9%)	295 (77.6%)	311 (82.1%)	0.056
Statin	377 (99.5%)	379 (99.7%)	379 (100%)	0.554

BMI: body mass index; SBP: systolic blood pressure; DBP: diastolic blood pressure; CAD: coronary artery disease; PT: prothrombin time; APTT: activated partial thromboplastin time; TnI: troponin I; CK: creatine kinase; CK-MB: creatine kinase-MB; LDL-C: low-density lipoprotein cholesterol; FAR: fibrinogen-to-albumin ratio; LVEF: left ventricular ejection fraction; NSTEMI: non-ST elevation myocardial infarction; ACEI: angiotensin-converting enzyme inhibitor; ARB: angiotensin receptor blocker. Data are presented as the mean ± SD, median (25th-75th interquartile range), or *n* (%).

**Table 2:** Independent risk factors for severe CAD by multivariate logistic regression analysis.

Variable	*β*	SE	Wald	OR	95% CI	*P* value
Age	0.020	0.006	9.823	1.020	1.007~1.033	0.002
Female	-0.328	0.134	5.972	0.720	0.554~0.937	0.015
Hypertension	0.360	0.146	6.070	1.434	1.076~1.909	0.014
Diabetes mellitus	0.484	0.135	12.872	1.623	1.246~2.114	<0.001
TnI	0.005	0.031	0.023	1.005	0.946~1.067	0.881
LVEF	-0.020	0.006	11.768	0.980	0.969~0.991	0.001
NSTEMI	0.845	0.156	29.268	2.329	1.714~3.163	<0.001
FAR	0.058	0.027	4.513	1.060	1.005~1.118	0.034

TnI: troponin I; LVEF: left ventricular ejection fraction; NSTEMI: non-ST elevation myocardial infarction; FAR: fibrinogen-to-albumin ratio.

**Table 3:** MACE incidences during the follow-up period.

Variable	Group 1 (*n* = 374)	Group 2 (*n* = 373)	Group 3 (*n* = 376)	*P* value
30 days				
MACE	9 (2.4%)	19 (5.1%)	27 (7.2%)	0.010
All-cause mortality	0	0	0	—
Myocardial reinfarction	2 (0.5%)	2 (0.5%)	3 (0.8%)	0.875
TVR	7 (1.9%)	18 (4.8%)	25 (6.6%)	0.006
6 months				
MACE	17 (4.5%)	36 (9.7%)	45 (12.0%)	0.001
All-cause mortality	1 (0.3%)	2 (0.5%)	1 (0.3%)	0.878
Myocardial reinfarction	2 (0.5%)	6 (1.6%)	11 (2.9%)	0.039
TVR	14 (3.7%)	32 (8.6%)	35 (9.3%)	0.006
1 year				
MACE	30 (8.0%)	53 (14.2%)	63 (16.8%)	0.001
All-cause mortality	1 (0.3%)	6 (1.6%)	3 (0.8%)	0.133
Myocardial reinfarction	6 (1.6%)	9 (2.4%)	15 (4.0%)	0.120
TVR	25 (6.7%)	45 (12.1%)	49 (13.0%)	0.010

MACE: major adverse cardiovascular event; TVR: target vessel revascularization.

**Table 4:** Univariate and multivariate Cox regression analysis for predictors of MACE.

	Univariate analysis			Multivariate analysis		
	HR	95% CI	*P* value	HR	95% CI	*P* value
30-day MACE						
Female	0.407	0.210~0.787	0.008	0.442	0.227~0.858	0.016
BMI	1.143	1.060~1.232	<0.001	1.148	1.061~1.242	0.001
NSTEMI	3.024	1.779~5.142	<0.001	2.235	1.256~3.980	0.006
TnI	1.040	0.970~1.115	0.268			
CK-MB	1.009	1.004~1.014	0.001	1.005	1.000~1.011	0.064
FAR	1.142	1.050~1.243	0.002	1.095	1.011~1.186	0.025
6-month MACE						
BMI	1.087	1.025~1.152	0.005	1.082	1.019~1.149	0.010
NSTEMI	3.065	2.058~4.564	<0.001	2.544	1.678~3.856	<0.001
TnI	1.050	1.003~1.099	0.038	0.984	0.914~1.060	0.675
CK-MB	1.008	1.003~1.013	0.002	1.004	0.997~1.010	0.241
FAR	1.120	1.047~1.197	0.001	1.076	1.009~1.147	0.026
1-year MACE						
BMI	1.059	1.009~1.112	0.020	1.061	1.010~1.115	0.018
NSTEMI	2.472	1.773~3.448	<0.001	2.001	1.392~2.878	<0.001
TnI	1.042	1.000~1.086	0.049	0.979	0.915~1.048	0.546
CK-MB	1.008	1.004~1.012	<0.001	1.005	1.000~1.010	0.050
FAR	1.113	1.053~1.177	<0.001	1.080	1.022~1.141	0.006

MACE: major adverse cardiovascular event; BMI: body mass index; NSTEMI: non-ST elevation myocardial infarction; TnI: troponin I; CK-MB: creatine kinase-MB; FAR: fibrinogen-to-albumin ratio.

**Table 5:** Evaluation of predictive models for MACE.

	C-statistic	NRI	*P*	IDI	*P*
30 days					
FAR	0.628 (0.560~0.696)				
∗Established risk factors	0.706 (0.633~0.780)	Ref.		Ref.	
Established risk factors + FAR	0.720 (0.647~0.793)	0.219 (-0.080~0.315)	0.084	0.005 (-0.002~0.022)	0.172
6 months					
FAR	0.609 (0.556~0.661)				
∗∗Established risk factors	0.650 (0.590~0.710)	Ref.			
Established risk factors + FAR	0.668 (0.612~0.725)	0.155 (-0.037~0.263)	0.070	0.003 (-0.001~0.014)	0.222
1 year					
FAR	0.593 (0.549~0.637)				
∗∗∗Established risk factors	0.611 (0.561~0.661)	Ref.		Ref.	
Established risk factors + FAR	0.632 (0.585~0.680)	0.136 (0.005~0.211)	0.044	0.006 (0.000~0.019)	0.042

FAR: fibrinogen-to-albumin ratio; NRI: net reclassification improvement; IDI: integrated discrimination improvement; MACE: major adverse cardiovascular event; ∗Established risk factors included female, BMI and NSTEMI; ∗∗Established risk factors included BMI and NSTEMI; ∗∗∗Established risk factors included BMI and NSTEMI.

## Data Availability

The datasets used and/or analyzed during the present study are available from the corresponding author on reasonable request.
